# Development and validation of a CT-texture analysis nomogram for preoperatively differentiating thymic epithelial tumor histologic subtypes

**DOI:** 10.1186/s40644-020-00364-5

**Published:** 2020-12-11

**Authors:** Caiyue Ren, Mingli Li, Yunyan Zhang, Shengjian Zhang

**Affiliations:** 1 Department of Nuclear Medicine, Shanghai Proton and Heavy Ion Center, Shanghai, 201315 China; 2 Shanghai Engineering Research Center of Proton and Heavy Ion Radiation Therapy, 4365 Kangxin Road, Shanghai, 201315 China; 3 Department of Radiology, Shanghai Proton and Heavy Ion Center, Shanghai, 201315 China; 4 Department of Radiology, Fudan University Shanghai Cancer Center, Shanghai, 200032 China

**Keywords:** Thymic epithelial tumor, Classification, Nomogram, Computed tomography, X-ray, Texture analysis

## Abstract

**Background:**

Thymic epithelial tumors (TETs) are the most common primary tumors in the anterior mediastinum, which have considerable histologic heterogeneity. This study aimed to develop and validate a nomogram based on computed tomography (CT) and texture analysis (TA) for preoperatively predicting the pathological classifications for TET patients.

**Methods:**

Totally TET 172 patients confirmed by postoperative pathology between January 2011 to April 2019 were retrospectively analyzed and randomly divided into training (*n* = 120) and validation (*n* = 52) cohorts. Preoperative clinical factors, CT signs and texture features of each patient were analyzed, and prediction models were developed using the least absolute shrinkage and selection operator (LASSO) regression. The performance of the models was evaluated and compared by the area under receiver-operator characteristic (ROC) curve (AUC) and the DeLong test. The clinical application value of the models was determined via the decision curve analysis (DCA). Then, a nomogram was developed based on the model with the best predictive efficiency and clinical utility and validated using the calibration plots.

**Results:**

Totally 87 patients with low-risk TET (LTET) (types A, AB, B1) and 85 patients with high-risk TET (HTET) (types B2, B3, C) were enrolled in this study. We separately constructed 4 prediction models for differentiating LTET from HTET using clinical, CT, texture features, and their combination. These 4 prediction models achieved AUCs of 0.66, 0.79, 0.82, 0.88 in the training cohort and 0.64, 0.82, 0.86, 0.94 in the validation cohort, respectively. The DeLong test and DCA showed that the Combined model, consisting of 2 CT signs and 2 texture parameters, held the highest predictive efficiency and clinical utility (*p* < 0.05). A prediction nomogram was subsequently developed using the 4 independently risk factors from the Combined model. The calibration curves indicated a good consistency between the actual observations and nomogram predictions for differentiating TET classifications.

**Conclusion:**

A prediction nomogram incorporating both the CT and texture parameters was constructed and validated in our study, which can be conveniently used for the preoperative individualized prediction of the simplified histologic subtypes in TET patients.

## Background

Thymic epithelial tumors (TETs), which are the most common primary tumors in the anterior mediastinum, are well-known for heterogeneity in the oncologic and biologic behaviors [1]. According to the morphology of epithelial cells and the lymphocyte-to-epithelial cell ratio, the World Health Organization (WHO) classification classified TETs into six subtypes (thymoma: types A, AB, B1, B2, and B3; thymic carcinoma: type C) in 2015, which is recognized as the basis of the clinical decision making and an independent prognostic factor in TETs [2–4]. Previous studies have shown that the tumor invasiveness of each subtype increases in turn, and patients with low-risk TET (LTET) (types A, AB, B1) usually have more chances to be completely resected, lower tumor recurrence rate and higher survival rate than ones with high-risk TET (HTET) (types B2, B3, C) [5–7]. Moreover, HTET patients can benefit more from adjuvant treatment than LTET patients [8]. Thus, preoperative knowledge of histologic classifications can provide valuable information for treatment decision making and prognostic evaluation in TET patients.

Computed tomography (CT) is widely recognized as the main imaging method for the diagnosis, differentiation, and evaluation of curative effect in TET patients due to its convenient operation, good image quality, moderate price, and fewer contraindications [9]. The signs on CT images, such as the tumor size, location, as well as the presence of pericardium or pleural effusion and distant metastases, are helpful to preliminarily judge the invasiveness of TETs [10–12]. However, they are limited for further accurately assessment of tumor heterogeneity or differentiation of its histological subtypes [13].

Texture analysis (TA) based on conventional medical images has been applied in the quantitative assessment of tumor heterogeneity by analyzing the distribution and relationship of pixel or voxel gray levels in the lesion area [14, 15]. Previous studies have revealed that TA, as a non-invasive imaging tool, has great potential in predicting pathologic features, response to therapy and prognosis of head and neck cancer, rectal cancer, et.al [16, 17]. CT quantitative TA also has been used in assessing anterior mediastinal lesions, including in distinguishing TET and non-TET diseases, estimating TET’s subtypes, grades and stages, et.al, and shown good diagnostic performance [18–20]. However, only texture parameters extracted from CT images were analyzed in the above studies, whether the prediction performance based on texture analysis can be further improved by combining CT signs is an interesting problem that requires investigation.

Hence, this study aimed to develop and validate a nomogram consisting of CT morphological features and texture parameters for differentiating the simplified histologic subtypes in patients with TET.

## Methods

### Patients

We conducted a retrospective analysis of records from patients with TET who were diagnosed by curative surgical resection between January 2011 to April 2019 at two cancer centers: Shanghai Proton and Heavy Ion Center (institution A) and Fudan University Shanghai Cancer Center (institution B). This retrospective study was approved by the ethics committees of these two institutions, and the requirement for informed consent was waived. The inclusion criteria included the following: 1) underwent standard contrast-enhanced CT at the above two institutions less than 14 days before surgery; 2) received radical surgery at institution B; 3) information of postoperative pathologically confirmed TET histologic subtypes available. The exclusion criteria included the following: 1) previous history of malignant tumors; 2) anti-tumor therapy before CT examination; 3) poor image quality affects lesion segmentation.

In total, 172 patients were enrolled and analyzed (95 males and 77 females; mean age, 54.56 ± 10.67 years; range, 24–77 years). Patients were divided into a training cohort (*n* = 120) and a validation cohort (*n* = 52) after simple randomization at a ratio of 7 to 3. Baseline data pertaining to the demographics of each patient, including gender, age, symptom was reviewed and recorded.

### CT images acquisition and analysis

Patients generally underwent contrast-enhanced CT of the entire thorax according to the standard clinical scanning protocols (tube voltage, 120 kV; tube current, 200 mA; pitch, 1.0; imaging matrix, 512 × 512; and reconstructed slice thickness, 1.0 mm) on the 32- or 64-slices Siemens Sensation System (Siemens Medical System, Forchheim, Germany). All CT scans were reconstructed into slices of 1-mm thickness and interval using a kernel that was suitable for interpreting mediastinal structures. Patients were in a supine position, and the scan range included all lesion areas. After the plain CT, a total of 80–120 mL (1.5 mL/kg) of iodinated contrast material (Ultravist 370, Bayer Schering Pharma, Berlin, Germany) was injected with a pump injector (Ulrich CT Plus 150, Ulrich Medical, Ulm, Germany) at a flow rate of 3 mL/s into the antecubital vein. The enhanced scan started 35 s after the injection of contrast media. The images were uploaded to the picture archiving and communication system (PACS) (Carestream, Ontario, Canada).

Two radiologists (reader 1: CY.R, with 3 years of experience in CT diagnosis, now working in the Department of Nuclear Medicine; reader 2: YY.Z, a radiologist who has 14-years working experience) assessed the following CT morphological features of each lesion without knowing the exact TET pathologic subtypes by consensus: tumor size (the longest diameter measured by the largest cross-section of the mass and the shortest diameter perpendicular to it), location (unilateral or cross midline), shape (regular or irregular), boundary (smooth or rough), density (the presence of calcification, cystic necrosis), the degree of enhancement compared to that of the chest wall muscle (mild enhancement: less than that of chest wall muscle; moderate enhancement: equal to that of chest wall muscle; obvious enhancement: higher than that of chest wall muscle), mediastinal fat line that means the fat planes between the tumor and adjacent mediastinal structures such as pericardium or great vessels (clear, unclear), pericardium or pleural effusion and metastasis (present or absent).

### Tumor segmentation and texture feature extraction

**Fig. 1:**
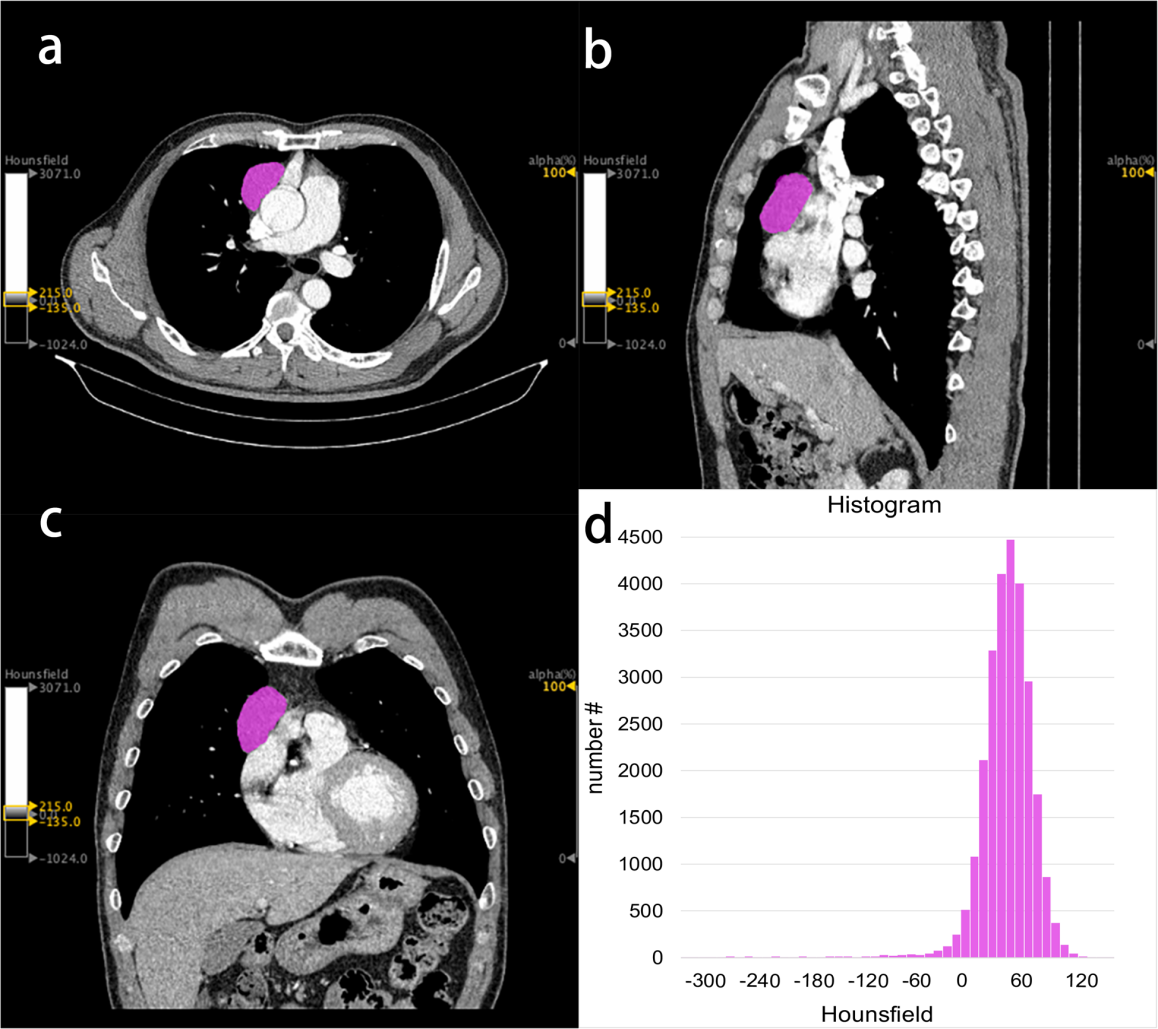
Chest enhanced CT images of a 53-years-old man with type B1 thymic epithelial tumor (low-risk TET). Texture features are extracted from the primary tumor area (purple overlay). **a** transverse section; **b** median sagittal section; **c** coronal section; **d** histogram

Tumor segmentation and feature extraction were performed using the LIFEx (version 5.10, www.lifexsoft.org) package [21]. The above two radiologists selected the largest slice of the tumor at three-dimensional (3D) images to delineate the region of interest (ROI) by consensus (Fig. 1 a-d). The ROI selection should include all tumors and avoid blood vessels, calcification and gas.

A total of 43 texture features were extracted automatically from the ROI [22], including 2 shape features, 9 first-order histogram features, 7 Gray-Level Co-occurrence Matrix (GLCM) features, 11 Gray-Level Run Length Matrix (GLRLM) features, 3 Neighboring Gray-Level Dependence Matrix (NGLDM) features and 11 Gray-Level Zone Length Matrix (GLZLM) features. The details of texture features were described in supplementary data (Table A).

### Statistical analysis

Statistical analysis was performed in R (version 3.6.0, http://www.r-project.org). A two-tailed *p* value of < 0.05 indicated statistical significance. The Mann-Whitney *U* test was used to assess the differences in continuous variables, whereas the χ^2^ test was used for categorical variables. Intra- and interclass correlation coefficients (ICCs) were used to evaluate the consistency and reproducibility of the intra- and inter-observer agreements of the feature extractions. An ICC greater than 0.75 indicated good consistency.

### Feature selection and prediction model establishment

Univariate analysis was applied to the clinical, CT, and texture features to identify the relevant predictors of the TET subtypes using Pearson’s correlation test in the training cohort. Multivariate analysis was performed by the least absolute shrinkage and selection operator (LASSO) regression with 10-fold cross-validation, which was used to select the most useful features [23, 24]. The prediction models for differentiating LTET from HTET were developed by the linear fusion of the selected features weighted by their coefficients, with prediction scores (Pre-scores) of each model calculated for each patient.

### Prediction performance and clinical utility of prediction models

The performance of the models was evaluated by the area under the receiver-operator characteristic (ROC) curve (AUC) and compared by the DeLong test. The AUC with 95% confidence interval (CI), sensitivity, specificity and accuracy were calculated for each model. The clinical application value of the prediction models was determined and compared through the decision curve analysis (DCA) by quantifying the net benefit to the patient under different threshold probabilities in the queue.

### Development and validation of a nomogram

To provide a visually quantitative tool to predict the histologic subtypes for TET patients, we develop a nomogram based on the prediction model with the highest AUC value and clinical utility on the training cohort. The calibration curves were plotted to assess the calibration of the nomogram by bootstrapping (1000 bootstrap resamples) based on internal (training cohort) and external (validation cohort) validity.

## Results

### Clinical and demographic characteristics

Totally 172 TET patients comprising of 87 LTET (n [type A] = 6; n [type AB] = 66; n [type B1] = 15) and 85 HTET (n [type B2] = 41; n [type B3] = 23; n [type C] = 21) were enrolled in this study. The patients’ clinical and demographic characteristics are summarized and compared in Table 1. The patient’s sex and age were highly related to the discrimination of the two subtypes (*p* < 0.05, respectively). There are no significant differences in the symptom between the LTET and HTET groups according to the univariate analysis in either the training or validation cohorts (*p* > 0.05, respectively), consistent with the report [25]. The long and short diameters (mean ± SD) of tumors in the training cohort were 51.61 ± 23.21 mm, 35.48 ± 16.44 mm in LTET; and 46.58 ± 17.46 mm, 29.85 ± 12.52 mm in HTET, respectively. The short diameter of LTET was significantly greater than that of HTET (*p* < 0.05), conversely, there was no statistical significance in long diameter between the LTET and HTET groups (*p* > 0.05).

**Table 1:** Clinical and demographic characteristics of TET patients

Characteristics	Training cohort		*p-*value	Validation cohort		*p*-value
	LTET (*n* = 61)	HTET (*n* = 59)		LTET (*n* = 26)	HTET (n = 26)	
Sex (%)						
Male	28 (45.90%)	38 (64.41%)	0.04	11 (42.31%)	18 (69.23%)	0.03
Female	33 (54.10%)	21 (35.59%)		15 (57.69%)	8 (30.77%)	
Age (mean ± SD, years)	56.44 ± 9.48	51.76 ± 12.08	0.02	56.50 ± 8.30	54.53 ± 11.02	0.04
Symptom (%)						
+	25 (40.98%)	30 (50.85%)	0.28	12 (46.15%)	16 (61.54%)	0.57
-	36 (59.02%)	29 (49.15%)		14 (53.85%)	10 (38.46%)	

Note: *LTET* low-risk thymic epithelial tumor, *HTET*, high-risk TET; SD standard deviation; *p*-values were the results of univariable association analyses of each characteristic

### Feature selection and prediction model establishment

A total of 12 CT signs and 43 texture features were extracted from 172 TET patients’ enhanced CT images, and the agreements between the two radiologists (readers 1, 2) were excellent for texture features (all ICCs > 0.85, *p* < 0.05). Thus, the mean measurement values of the two radiologists were used for further analysis.

**Fig. 2:**
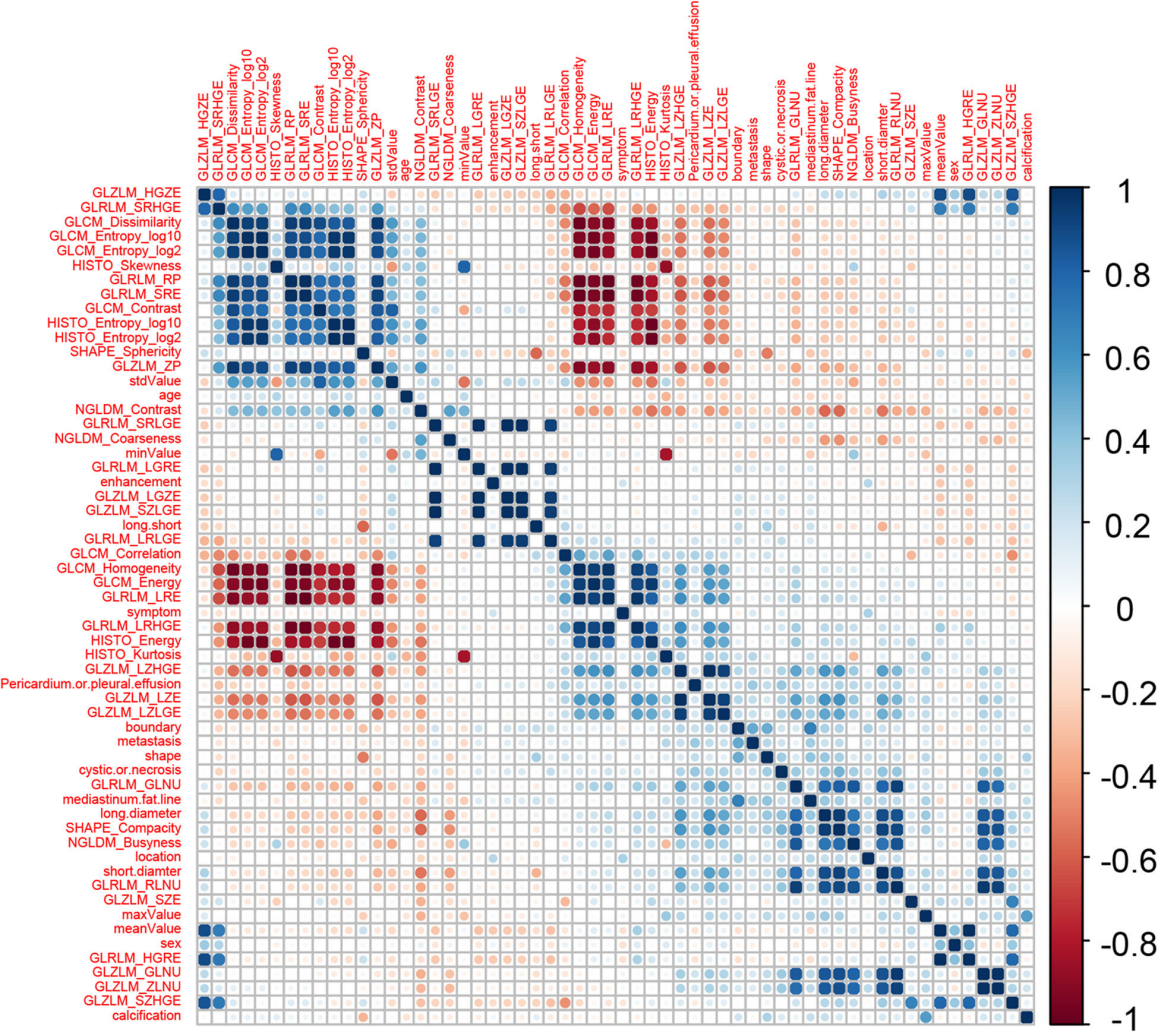
The cross-correlation matrix for covariates. Blue represents positive correlation and red represents negative correlation. The depth of color indicates the intensity of the correlation between covariates. The darker the color, the higher the correlation is

**Fig. 3:**
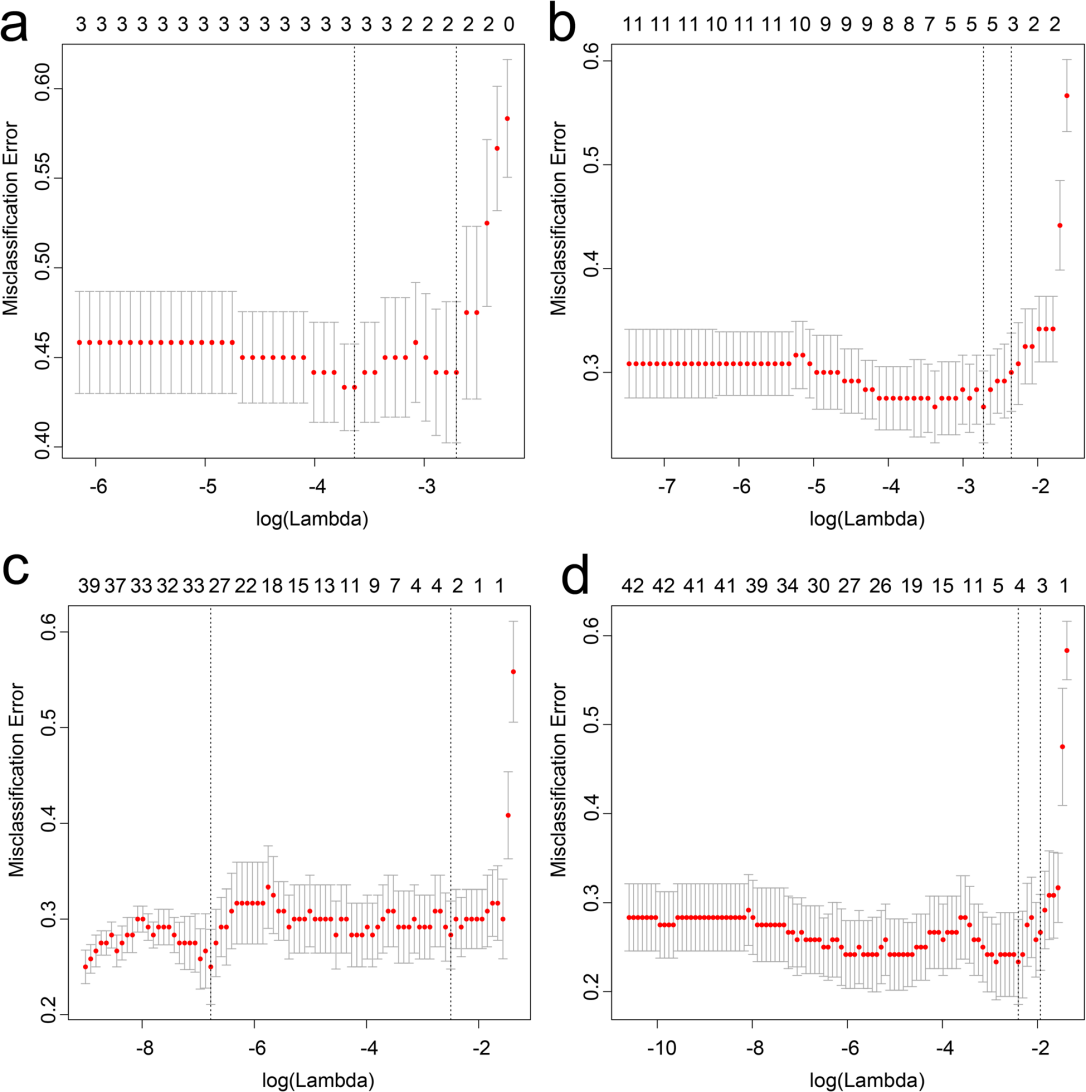
Features selection for the prediction models by LASSO regression. Tuning parameter (λ) selection used 10-folds cross-validation. The X-axis shows log (λ), and the Y-axis shows the model misclassification rate. The 2, 3, 3,4 features with non-zero coefficients are indicated with the optimal λ values of 0.07, 0.10, 0.08, 0.10 for Clinical model (**a**), CT model (**b**), TA model (**c**), Combined model (**d**), respectively

The cross-correlation matrixes showed that there were multiple complex cross-correlations among these parameters (Fig. 2). For differentiating LTET from HTET, 4 independent prediction models were built separately based on the selected clinical, CT, texture parameters, and their combination by LASSO regression in the training cohort (Fig. 3 a-d). The Pre-scores of each model for each patient were calculated using the following formulas:

- Pre-scores (Clinical model) = 0.83–0.20*sex - 0.01*age;
- Pre-scores (CT model) = − 0.16 - 0.01*short diameter + 0.49*boundary + 0.63*mediastinum fat line;
- Pre-scores (TA model) = 3.01–0.04*meanValue - 0.62*SHAPE_Sphericity − 0.03*NGLDM_Busyness;
- Pre-scores (Combined model) = 1.67 + 0.39*boundary + 0.46*mediastinum fat line − 0.03*meanValue - 0.03*NGLDM_Busyness.

HTET patients generally had higher Pre-scores for all prediction models than LTET patients in both the training and validation cohorts (*p* < 0.05, respectively) (Table 2).

**Table 2:** Pre-scores of prediction models and their compositions of TET patients in the training cohort

Short diameter (mean ± SD, mm)	LTET (*n* = 61)	HTET (*n* = 59)	*p*-value
	35.48 ± 16.44	29.85 ± 12.52	0.04
Boundary (%)			
Smooth	53 (86.89%)	30 (50.85%)	0.00
Rough	8 (13.11%)	29 (49.15%)	
Mediastinum fat line (%)			
clear	45 (73.77%)	20 (33.90%)	0.00
unclear	16 (26.23%)	39 (66.10%)	
MeanValue	68.63 (56.52, 78.55) ^a^	48.84 (40.82, 58.18) ^a^	0.00
SHAPE_Sphericity	0.95 (0.93, 0.97) ^a^	0.93 (0.90, 0.96) ^a^	0.01
NGLDM_Busyness	1.13 (0.36, 3.64) ^a^	0.91 (0.43, 1.63) ^a^	0.01
Pre-scores (Clinical model)	−0.11 (−0.23, 0.06) ^a^	−0.01 (−0.11, 0.13) ^a^	0.00
Pre-scores (CT model)	− 0.37 (− 0.50, − 0.22) ^a^	0.29 (− 0.33, 0.63) ^a^	0.00
Pre-scores (TA model)	−0.53 (− 0.85, 0.02) ^a^	0.32 (− 0.01, 0.76) ^a^	0.00
Pre-scores (Combined model)	− 0.53 (− 0.99, − 0.05) ^a^	0.52 (0.03, 0.84) ^a^	0.00

*Note*: *NGLDM* Neighboring Gray-Level Dependence Matrix, *CT* computed tomography, *TA* texture analysis; ^a^Values refer to median (interquartile range (IQR))

### Prediction performance and clinical utility of prediction models

**Fig. 4:**
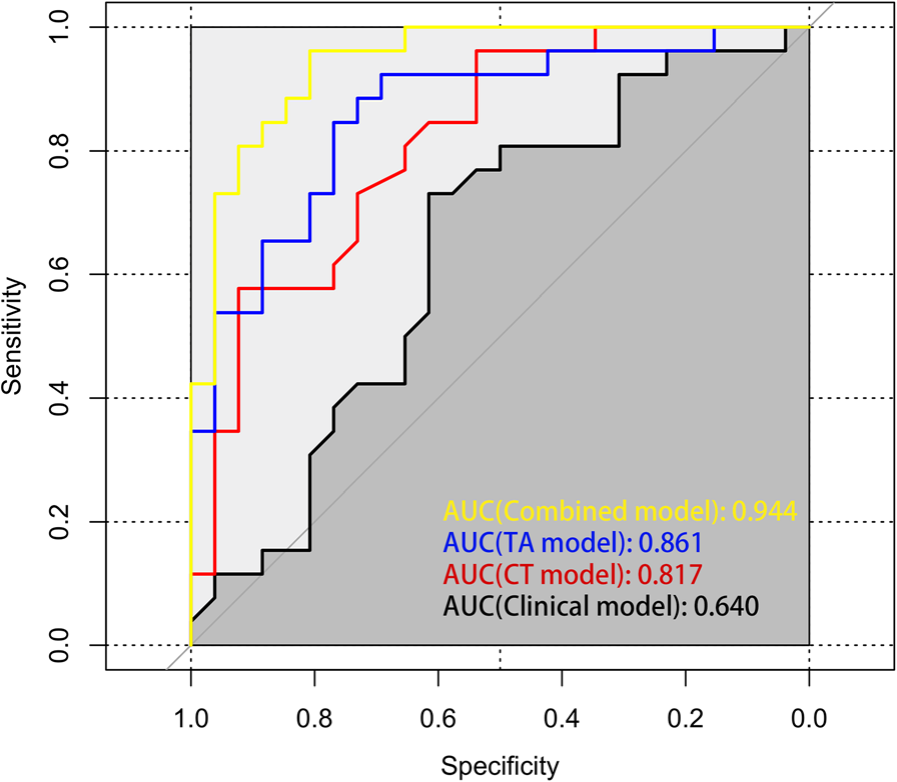
ROC curve analysis of the prediction models in the validation cohort

The performance of these 4 models to discriminate LTET from HTET is shown in Table 3 and Fig. 4. The discriminant capacity of Clinical model, CT model, TA model, and Combined model increased in turn, which indicated that the Combined model presented the optimal discrimination and best predictive accuracy with the highest AUC and accuracy in both the training cohort (AUC [95% CI] = 0.88 [0.81–0.94], accuracy = 79.2%) and the validation cohort (AUC [95% CI] = 0.94 [0.89–0.98], accuracy = 86.5%) (Table 3).

**Table 3:** Prediction performance of the 4 prediction models

**Training cohort**	**AUC**	**95% CI**	**Sensitivity (%)**	**Specificity (%)**	**Accuracy (%)**
Clinical model	0.66	0.56–0.75	81.4	45.9	59.2
CT model	0.79	0.70–0.87	66.1	83.6	71.7
TA model	0.82	0.74–0.89	94.9	60.7	75.0
Combined model	0.88	0.81–0.94	93.2	67.2	79.2
**Validation cohort**	**AUC**	**95% CI**	**Sensitivity (%)**	**Specificity (%)**	**Accuracy (%)**
Clinical model	0.64	0.49–0.79	73.1	61.5	57.7
CT model	0.82	0.70–0.93	96.2	53.8	69.2
TA model	0.86	0.76–0.96	88.5	73.1	75.0
Combined model	0.94	0.89–0.98	96.2	80.8	86.5

*Note*: *AUC* area under the receiver operating curve, *95% CI* 95% confidence interval

**Fig. 5:**
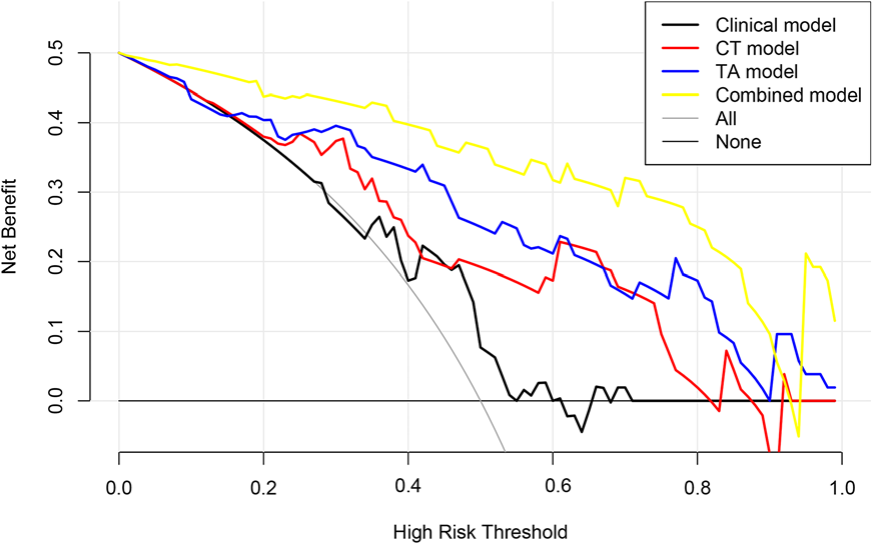
DCA for the prediction models. The X-axis represents the threshold probability. The Y-axis represents the net benefit. The grey and black thin lines represent the hypothesis that all TET patients are high-risk or low-risk, respectively. The higher curve at any given threshold probability is the optimal prediction to maximize net benefit

The DCA also showed that the clinical application value of these 4 prediction models increased in turn, which indicated that the Combined model, incorporating 2 CT morphological features and 2 texture parameters, was a most reliable clinical treatment tool for predicting the histologic subtypes in TET patients when the threshold probability was between 0.02 and 0.91 (Fig. 5).

### Development and validation of a nomogram

**Fig. 6:**
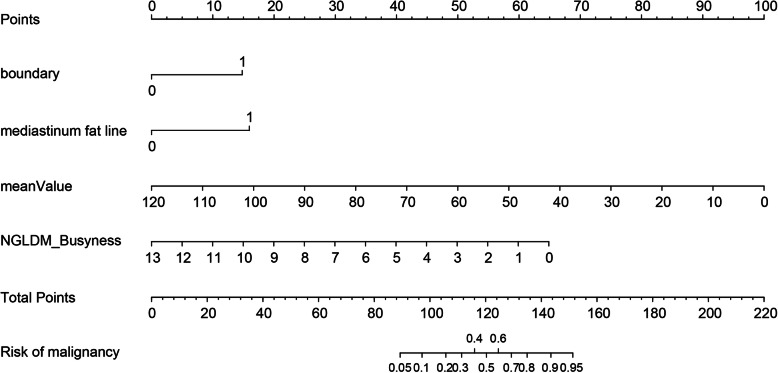
Developed prediction nomogram in the training cohort. The probability of each predictor can be converted into scores according to the first scale “Points” at the top of the nomogram. After adding up the corresponding prediction probability at the bottom of the nomogram is the malignancy of the tumor

**Fig. 7:**
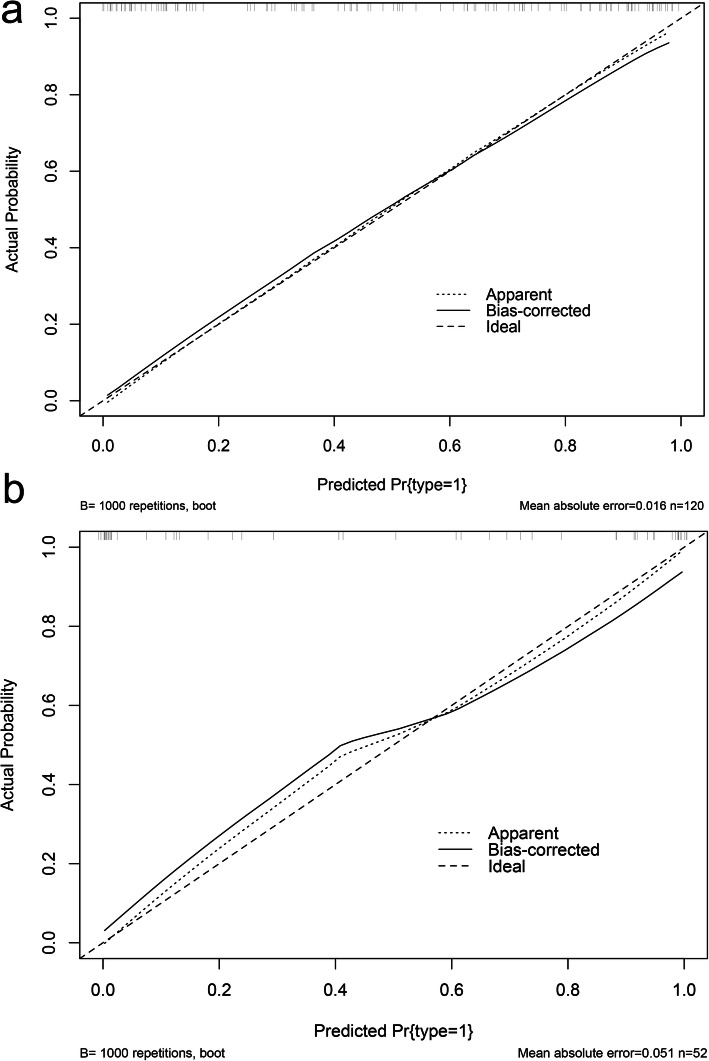
Calibration curves of the nomogram in training (**a**) and validation (**b**) cohorts. The X-axis represents the predicted malignancy probability estimated by the nomogram whereas the Y-axis represents the actual observed rates of HTET. The solid line represents the ideal reference line that predicted TET malignant corresponds to the actual outcome, the short-dashed line represents the apparent prediction of nomogram, and the long-dashed line represents the ideal estimation

According to the above results, we generated a nomogram based on the parameters of the Combined model for visualization (Fig. 6). The calibration curves of the nomogram for the probability of HTET demonstrated good agreements between the nomogram and the actual observations in both the two cohorts (*p* > 0.05, respectively) (Fig. 7).

## Discussion

In the present study, we developed 4 independent prediction models to differentiate the TET pathologic subtypes, and constructed a quantitative nomogram based on the model which held the highest efficiency and clinical utility. This nomogram was validated for the preoperative individualized prediction of the classifications in TET patients.

In terms of the clinical characteristics and CT signs, although the correlation between patient’s gender, age and tumor invasiveness is still controversial [2, 4, 12], our study showed that an older female TET patient with a bigger tumor size, a smoother boundary and a clearer mediastinum fat line indicated a lower probability that the tumor was malignant. In this study, the tumor size, boundary, and mediastinum fat line were significantly ​​associated with the malignant grades of TET. According to the general understanding, the larger the tumor, the more malignant it is [26]. However, the sizes in LTET were larger than that in HTET in both the training and validation cohorts (*p* values < 0.05, respectively). This may be related to the fact that the less aggressive the tumor is, the lighter clinical symptoms the patient has, which leads to a larger tumor volume when it is found. The boundary and mediastinum fat line of the tumor on CT images can reflect the mass invasiveness to a certain extent [27, 28]. There is usually a higher rate of the tumor invasiveness by directly extending to adjacent structures including vessels, pericardium, or lung, which is shown as a rough boundary and blurred or even disappeared fat line. The results of this study are consistent with previous researches.

In this study, the texture parameters of meanValue, sphericity, and NGLDM_Busyness of LTET patients were higher than HTET ones. Meanwhile, the TA model that composed of these 3 parameters held great individualized prediction for TET patients (AUCs = 0.82 [training cohort], 0.86 [validation cohort], respectively). MeanValue in the histogram, which represents the average value of ROI, reflects the degree of texture regularity: the higher the value, the more regular the texture is, that is, the lower the heterogeneity is. Yasaka K et al. also found that the meanValue was a significant parameter for differentiating HTET from LTET with AUC of 0.89 [18]. Sphericity is the shape feature of the tumor and has been proven as the most significant factor for discriminating histologic subtypes in TET patients [29]. Busyness is a parameter of NGLDM, which measures the spatial frequency of changes in intensity between nearby voxels of different grey-levels. The role of busyness in TETs has not been reported before, but it has been used to assess the tissue heterogeneity in glioma and lung cancer [30–32]. Heterogeneity is a recognized feature of the tumor that considered to be positively correlated with the tumor malignancy, which is of great clinical significance for effective personalized therapies [33, 34]. Previous studies also demonstrated that thymic carcinoma, type B2, and B3 thymomas are generally more heterogeneous than type A, AB, B1 thymomas [35–37]. The results of this study are consistent with the above reports.

This study also explored whether the prediction performance based on texture analysis could be improved by combination with conventional CT diagnosis. The Combined model developed in the present study, consisting of the boundary, mediastinum fat line, meanValue, and NGLDM_Busyness, was most advantageous than did the use of either them alone. The accuracy of the Combined model was also superior. The results of this study confirm the hypothesis and indicate that the heterogeneity of the tumor can be evaluated more comprehensively by combining with the macroscopic and internal characteristics of the tumor. In addition, we generated a nomogram based on the Combined model for facilitating clinical use, and recommend that a younger male patient with a smaller tumor size, a rougher boundary, an unclearer mediastinum fat line shown on preoperative enhanced CT images should have a more regular follow-up, and the progression also should be closely monitored. Besides, we suggest that patients with a higher-risk of TET, as screened by the nomogram, should be considered potential adjuvant therapy candidates to extend survival. The clinical application of this nomogram can reduce the cost of subsequent diagnosis, help develop more reasonable and effective treatment plans, and prevent patients from having a poor prognosis.

However, the present study had several limitations although the results were encouraging. First, the sample selection was biased in this retrospective study, and a prospective study is required to confirm and validate the prediction nomogram. Second, the texture analysis in this study was based on enhanced CT, which can increase the risk of adverse reactions of contrast media, such as acute hypersensitivity reactions [38]. Whether the use of plain CT or the combination of plain and enhanced CT will increase the predictive efficiency needs further study. Third, the tumor, node, metastasis (TNM) [39], or Masaoka [40] staging systems for TETs were not used in this study. Further study will be needed to reveal the relationship between texture features and TNM or Masaoka staging systems. Finally, this study only included TETs. Additional studies that include other tumors or tumor-like lesions in the anterior mediastinum for better characterization will be performed.

## Conclusions

A prediction nomogram incorporating both the CT morphological features and texture parameters was constructed and validated in our study, which was conveniently used to facilitate the preoperative individualized prediction of the simplified histologic subtypes in TET patients.

## Supplementary Information

**Additional file 1: Table A** Specific categories of texture parameters.

## Data Availability

Yes.

## Abbreviations

3D: Three-dimensional
AUC: The area under ROC curve
CI: Confidence intervals
CT: Computed tomography
DCA: Decision curve analysis
GLCM: Gray-Level Co-occurrence Matrix
GLRLM: Gray-Level Run Length Matrix
GLZLM: Gray-Level Zone Length Matrix
HTET: High-risk TET
ICCs: Intra- and interclass correlation coefficients
LASSO: Least absolute shrinkage and selection operator
LTET: Low-risk TET
NGLDM: Neighboring Gray-Level Dependence Matrix
PACS: Picture archiving and communication system
Pre-score: Prediction score
ROC: Receiver operating characteristic.
ROI: Region of interest.
TA: Texture analysis.
TET: Thymic epithelial tumor.
TNM: Tumor, node, metastasis.
WHO: World Health Organization.
