# Supplementary material for: Development and validation of a CT-texture analysis nomogram for preoperatively differentiating thymic epithelial tumor histologic subtypes
Source: Cancer Imaging. 2020 Dec 11;20:86. doi: 10.1186/s40644-020-00364-5 (PMC7731456; doi:10.1186/s40644-020-00364-5)
Supplement: Supplementary file 1 — Additional file 1: Table A Specific categories of texture parameters. [file 40644_2020_364_MOESM1_ESM.doc]

**Appendices**

| **Table A** Specific categories of texture parameters | | |
| --- | --- | --- |
| Type | Feature Name (Symbol/abbreviation) | Description |
| Shape | Sphericity (-) | Measures how spherical a Volume of Interest is. Shericity is equal to 1 for a perfect sphere. |
|  | Compacity (-) | Measures how compact the Volume of Interest is. |
| Histogram | SkewnessHisto (-) | Measures the asymmetry of the gray-level distribution in the histogram. |
|  | Kurtosis (-) | Measures whether the gray-level distribution is peaked or flat relative to a normal distribution. |
|  | Entropy_log10/log2 (Entropy H) | Measures the randomness of the distribution. |
|  | Energy (Energy H) | Measures the uniformity of the distribution. |
|  | MinValue (-) | Measures the minimum in the Volume of Interest. |
|  | MeanValue (-) | Measures the average in the Volume of Interest. |
|  | StdValue (-) | Measures the standard deviation in the Volume of Interest. |
|  | MaxValue (-) | Measures the maximum in the Volume of Interest. |
| GLCM (Gray-level co-occurrence matrix) | Homogeneity (-) | Measures the homogeneity of gray-level voxel pairs. |
|  | Energy (-) | Also called Uniformity or Second Angular Moment, measures the uniformity of gray- level voxel pairs. |
|  | Contrast (-) | Also called Variance or Inertia, measures the local variations in the GLCM. |
|  | Correlation (-) | Measures the linear dependency of gray-levels in GLCM. |
|  | Entropy_log10/log2 (-) | Measures the randomness of gray-level voxel pairs. |
|  | Dissimilarity (-) | Measures the variation of gray-level voxel pairs. |
| GLRLM (Grey level run length matrix) | Short-Run Emphasis (SRE) | SRE and LRE measure the distribution of the short or the long homogeneous runs in an image respectively. |
|  | Long-Run Emphasis (LRE) |  |
|  | Low Gray-level Run Emphasis (LGRE) | LGRE and HGRE measure the distribution of the low or high gray-level runs respectively. |
|  | High Gray-level Run Emphasis (HGRE) |  |
|  | Short-Run Low Gray-level Emphasis (SRLGE) | SRLGE and SRHGE measure the distribution of the short homogenous runs with low or high gray-levels respectively. |
|  | Short-Run High Gray-level Emphasis (SRHGE) |  |
|  | Long-Run Low Gray-level Emphasis (LRLGE) | LRLGE and LRHGE measure the distribution of the long homogeneous runs with low or high gray-levels respectively. |
|  | Long-Run High Gray-level Emphasis (LRHGE) |  |
|  | Gray-Level Non-Uniformity for run (GLNUr) | GLNUr and RLNU measure the non-uniformity of the gray-levels or the length of the homogeneous runs respectively. |
|  | Run Length Non-Uniformity (RLNU) |  |
|  | Run Percentage (RP) | Measures the homogeneity of the homogeneous runs. |
| NGLDM (Neighborhood gray-level different matrix) | Coarseness (-) | Measures the level of spatial rate of change in intensity. |
|  | Contrast (-) | Measures the intensity difference between neighboring regions. |
|  | Busyness (-) | Measures the spatial frequency of changes in intensity. |
